# Rethinking the Nature and Extent of Inductive Effects in Organic Compounds

**DOI:** 10.1021/acs.jchemed.6c00141

**Published:** 2026-05-14

**Authors:** Mark C. Elliott, Edwin C. Johnson, Kasimir P. Gregory, Colan. E. Hughes

**Affiliations:** † School of Chemistry, 2112 Cardiff University, Park Place, Cardiff CF10 3AT, U.K.; ‡ College of Science, Engineering and Environment, University of Newcastle, Callaghan, 2308 NSW, Australia; § School of Science and Technology, University of New England, Armidale, NSW 2351, Australia

**Keywords:** First-Year Undergraduate/General, Organic Chemistry, Physical Chemistry, Misconceptions/Discrepant Events, Carboxylic Acids, Covalent Bonding, Computational Chemistry

## Abstract

The inductive effect is generally taught as being transmitted through the σ-bond framework in organic molecules, diminishing with each bond. This historical position does not align with more recent studies, and we show that the inductive effect in neutral molecules is effectively limited to one bond. Evidence of onward transmission (e.g., ^13^C NMR chemical shifts) should be viewed with scepticism. In charged species, where an electron-donating functional group is coupled with a suitably disposed electron-withdrawing substituent, the effect is transmitted over more bonds. These examples should be considered as polarizability rather than ‘purely’ inductive. Examples that are commonly described as a through-space field effect are better explained by polarizability/orbital perturbation. This work presents a more complete view of the inductive effect and its interface with polarizability.

## Graphical abstract

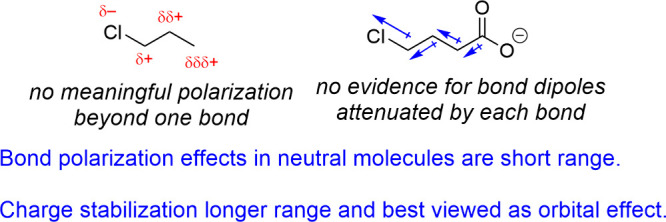

## Introduction

The inductive effect is one of the cornerstones of chemistry, introduced (but not named) by Lewis to explain properties such as the strength of acids and bases. In 1916, Lewis wrote that “we may consider acetic acid, in which one hydrogen is replaced by chlorine, H_2_ClCCOOH. The electrons, being drawn towards the chlorine, permit the pair of electrons joining the methyl and carboxyl groups to approach nearer to the methyl carbon. This pair of electrons, exercising therefore a smaller repulsion upon the other electrons of the hydroxyl oxygen, permit these also to shift in the same direction.”^1^

Ingold, in 1934,^2^ introduced the symbolic representation Cl←CH_2_←CH_2_←CH_3_ to indicate the sequential permanent polarization of bonds and stated that “it has been designated the inductive effect”.^3^ Wheland and Pauling^4^ attempted to quantify the charge transmission in aromatic systems using what is essentially Hückel theory. This paper introduced the notation of using “suitable δ’s upon the proper atoms” which has led to diagrams such as Figure 1. They also mention polarizability as a “small correction.” Due to limitations in the available quantum chemical techniques at the time, assumptions, rather than calculations, about the nature of charge transmission were invoked, and then parameters calculated based on these assumptions.

**Figure 1:**
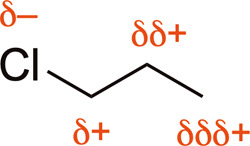
Typical textbook representation of transmission of the inductive effect through several bonds in a 1-chloropropane molecule.

The scope of the inductive effect has been broadened over the years and is now (IUPAC definition)^5^ “an experimentally observable effect (on rates of reaction, etc.) of the *transmission* of charge through a chain of atoms by electrostatic induction.” An earlier definition^6^ describes the inductive effect as “through bond transmission by successive polarization of the bonds between a dipolar or charged substituent and a reaction site. The effect is attenuated by each bond in a ratio described as the transmission coefficient.” There have been several attempts to determine the transmission coefficient,^7^ and the early pedagogy of the area has been summarized by Stock.^8^ These ideas are now standard, with diagrams such as Figure 1 appearing in textbooks^9–19^ to illustrate the transmission of inductive effects over several bonds.

Much of the pedagogy of the inductive effect derives from the acidity of carboxylic acids. Aqueous p*K*_a_ values for a selection of carboxylic acids^14,20–27^ are shown in Figure 2.

**Figure 2:**
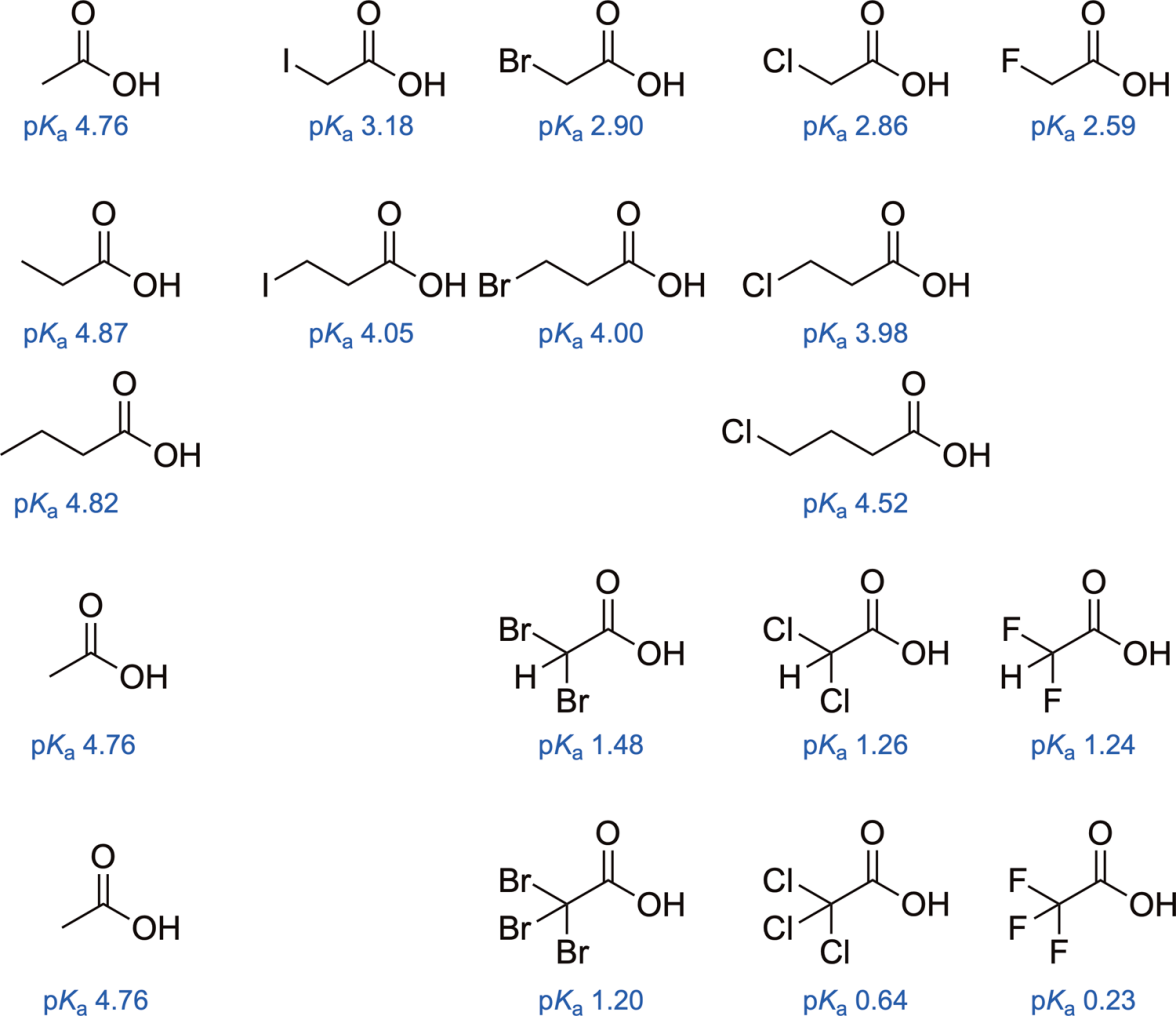
p*K*_a_ values for representative (halo)­carboxylic acids.

Several conclusions are commonly drawn from these data.

1. Halogenation renders a carboxylic acid more acidic.
2. A greater number of halogens exerts a larger effect.
3. The effect is larger for the most electronegative halogens.
4. The impact of the halogen is felt several bonds away, with closer halogens exerting a larger effect.

While points 1 and 2 are correct, point 3 is incorrect when gas phase acidity is considered and point 4 leads to assumptions about the nature of charge transmission that are not supported by the evidence. Chloroacetic acid is more acidic in the gas phase than fluoroacetic acid.^28^ Gas phase acidities are not available for many halocarboxylic acids. As such, we must often rely on computational data. Since their introduction in 1953,^29^ 4-substituted bicyclo[2.2.2]­octane-1-carboxylic acids (Figure 3) have been used for determination of inductive substituent parameters that are the foundation of physical organic chemistry.^7,30–34^ The Taft σ_I_ parameters of the halogens are given as F, 0.50; Cl, 0.47; Br, 0.45,^34^ which is in the expected inductive order. Exner and co-workers^35,36^ determined computational inductive parameters as F, 0.364; Cl, 0.444; Br, 0.482,^37^ so that the intrinsic enhancement of acidity of the carboxylic acid is actually Br > Cl > F.

**Figure 3:**
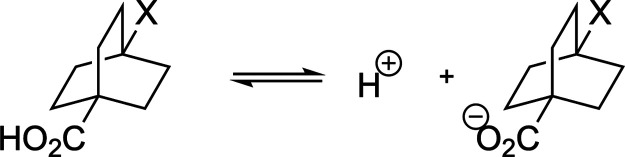
[2.2.2]­Bicyclooctane-1-carboxylic acids used to define inductive parameters for substituents ‘X’.

A recent study of gas-phase acidities in trihaloacetic acids show similar trends that again do not align with the textbook inductive model.^38^ It seems that the trends in solution p*K*_a_ data are significantly influenced by solvent effects, with the carboxylate anions containing the smaller halogens being better solvated. It is well established that the acidity of haloforms decreases in the order CHBr_3_ > CHCl_3_ > CHCF_3_, and the aqueous solution acidity of 4-halophenols also decreases in the order I > Br > Cl > F,^39^ both of which do not follow the expected inductive order but reflect the importance of polarizability.

In the present work, we explore a wider range of halocarboxylic acids to show that the above is general. We must therefore re-evaluate the role of the inductive effect in these systems.

Diagrams such as Figure 1 are used to show how the inductive effect is attenuated along an alkyl chain. From the initial report of Lewis, these ideas derive from experimental data such as carboxylic acid^40^ and alcohol acidity,^41–43^ and we will show that they do not mean that the inductive effect is attenuated by a specified factor with each additional C–C bond in carboxylic acids/carboxylate anions. Nor do they mean that the inductive effect in neutral molecules is transmitted in the same way.

NMR chemical shifts are sometimes used as evidence of transmission of the inductive effect.^44^ The ^13^C NMR chemical shift at C3 in pentane^45^ is higher than that at C2/C4. More importantly, C3 in pentane has a higher chemical shift than that in 1-chloropentane^45^ and 1,1,1-trichloropentane^46^ (Figure 4). It is difficult to reconcile these data with inductive effects of either alkyl groups^47^ or halogens, despite the latter two compounds appearing to give a show a trend consistent with an inductive effect that diminishes with each bond. Carbon chemical shift does not correlate directly with charge,^36,47^ and the γ-effect,^48^ attributed to van der Waals interactions,^49^ is routinely invoked to explain observations like this. We should be cautious using ^13^C chemical shift as a proxy for charge. The ^1^H chemical shift data presented by Cao and Cao^44^ show a significant effect on chemical shift within two bonds of a substituent, a much smaller effect at three bonds, and essentially no difference beyond this point.

**Figure 4:**
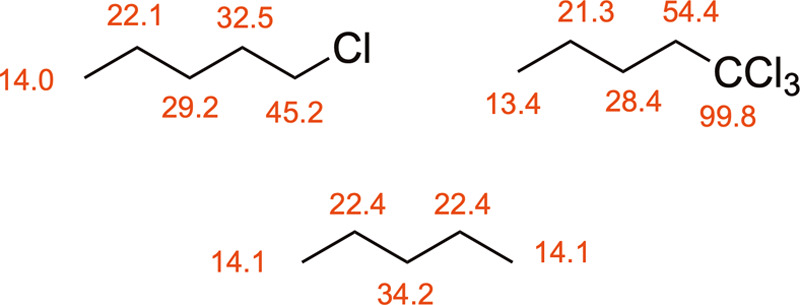
^13^C NMR chemical shifts for 1-chloropentane, 1,1,1-trichloropentane and pentane.

A more extreme example further illustrates the complexity of chemical shift. The carbocation carbon of the *t*-butyl group has a chemical shift of 335.2 ppm compared to 320.6 ppm in the 2-propyl carbocation^50^ despite the former being stabilized by electron-donation (hyperconjugation) from the additional methyl group. In essence, the chemical shift ‘ignores’ the p-orbital contribution to the overall electron-density, since the p-orbital has a nodal plane passing through the carbon nucleus.^51^

There is already evidence that the inductive effect is not transmitted along an alkyl chain in a neutral molecule.^52–56^ This appears not to be widely known and has had little impact on textbooks or on the pedagogy literature. In particular, Wiberg found (using QTAIM calculations) little onward transmission of the inductive effect in compounds such as fluorobutane.^57^ Nolan and Linck reached similar conclusions for NPA and AIM charges in fluoroalkanes and related compounds.^58^

In this work, we expand on previous studies to probe the inductive effect in a range of neutral molecules and halocarboxylic acids, to provide a more coherent description of the inductive effect and the related polarizability and field effects.

## Results and Discussion

### Computational Methodology

To probe the impact of substituents on the charge distribution within a molecule, we must use methods for the calculation of atomic charge. The ‘charge on an atom’ cannot be unambiguously defined.^59^ In previous work^60^ we benchmarked data based on the alignment of dipole moments from point charges with those from the overall density. However, some methods (e.g., CHELPG) provide good dipole moments, and yet might not be a good representation of atomic charge.^61^ Here, we reach conclusions that are consistent across a range of ‘good’ charge models. We will generally present NPA charges^62^ (using NBO 3.1 within Gaussian), although identical conclusions are reached using Hirshfeld^63^ and CM5 charges.^64^ Hirshfeld charges will be discussed in some cases, and data for all three charge models are in the Supporting Information. Since Nolan and Linck^58^ found that (for the type of systems investigated) NPA and QTAIM charges also correlate well, our conclusions are transferrable across a selection of well-regarded methods. We used DFT with the PBEh1PBE^65^ functional and a flexible orbital basis set (aug-cc-pVTZ^66–68^ ), using the Gaussian ‘09 software.^69^ This was deemed to be a reasonable compromise between accuracy and computational cost.

### Transmission of Charge in Neutral Molecules

#### Aliphatic Compounds

We first consider the charge distribution in pentane, 1-fluoropentane and 1,1,1-trifluoropentane to probe the limits of the inductive effect in neutral molecules. The data in Figure 5a show that fluorine exerts a significant effect on the carbon charge at C1 compared to pentane. There is essentially no effect at C3–C5, while C2 is electron-rich relative to pentane itself, directly contradicting the expectation of an effect transmitted along the chain attenuated by each bond. The effect at C2 due to fluorine is consistent with an electron-donating anomeric-type effect^70,71^ from the fluorine lone pairs^72^ (Figure 6). The orbital interaction is seen (24.8 kJ mol^–1^) in the NBO second order perturbation matrix. This might be a more significant inductive effect (−I) accompanied by a larger anomeric-type effect. The balance of the effects is discussed below.

**Figure 5:**
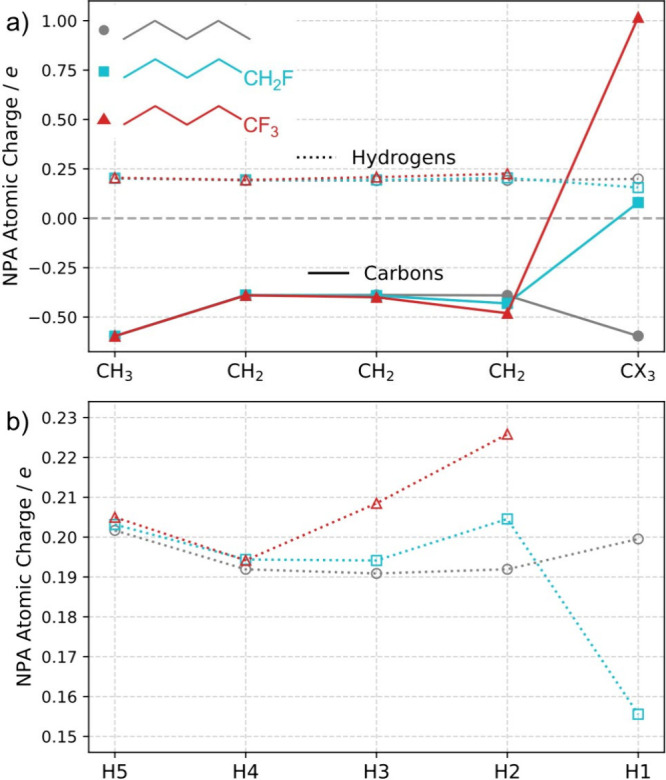
(a) NPA charges on hydrogen and carbon in pentane, 1-fluoropentane and in 1,1,1-trifluoropentane; (b) expansion of NPA charges on the hydrogen atoms along the pentane chains.

**Figure 6:**
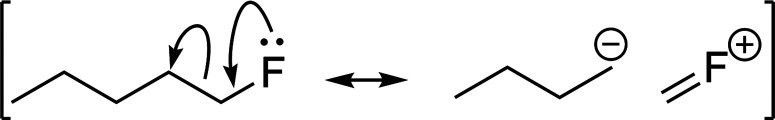
Hyperconjugation resonance forms representing n to π* anomeric-type interaction for 1-fluoropentane.

The hydrogen charges are expanded in Figure 5b, showing little difference between them. The H on C1 in 1-fluoropentane are calculated to be electron-rich compared to those in pentane, again consistent with hyperconjugation. This is not inconsistent with the higher ^1^H NMR chemical shifts of these carbon atoms, since charge-density at carbon is a better determining factor for the resonance frequency of an attached hydrogen atom.^73^ Our data are consistent with those of Wiberg^57^ and of Nolan and Linck.^58^

Data for pentane, 1,1,1-trifluoropentane, 1,1,1-trichloropentane and 1,1,1-tribromopentane are given in Figure 7. According to NPA charges, at C1 the effect is clearly F > Cl > Br as expected. At C2, the effect is H > Cl > F > Br, with minimal difference between the halogens. We might anticipate the +R (electron-donating hyperconjugation) effect and any inductive effect at this position to decrease in the order F > Cl > Br. Therefore, we could rationalize the similar charge at C2 with CF_3_ compared to CBr_3_ to be due to a larger inductive effect offset by a larger hyperconjugation effect. Chlorine and bromine behave similarly to fluorine in respect of the charges at H3–H4. At C3–C5 there is no meaningful effect at carbon.

**Figure 7:**
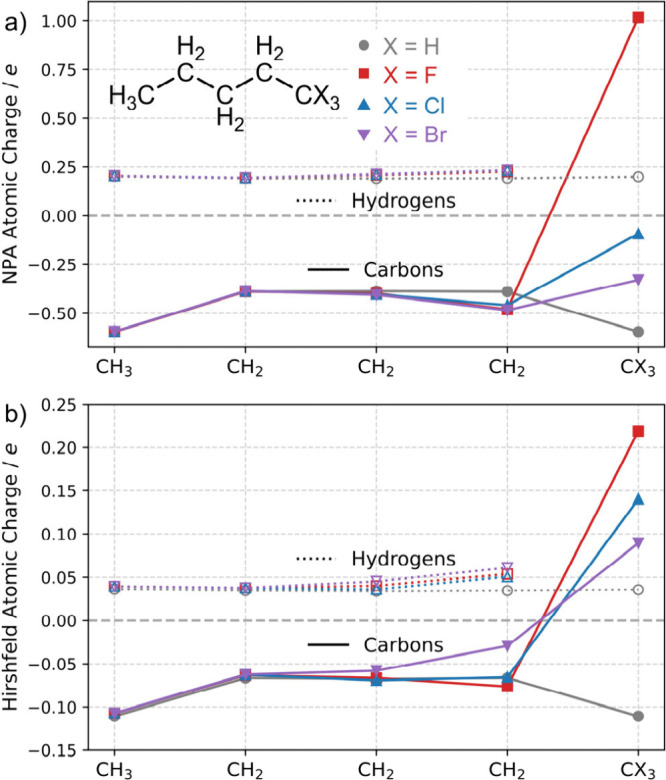
(a) NPA and (b) Hirshfeld atomic charges of trihalopentanes. Carbon (solid lines) and hydrogen (dotted lines).

Hirshfeld charges (Figure 7b) show small differences, with the effect at C2 in the order Br > H ≈ Cl > F at this site. On this basis, we would consider the hyperconjugation effect from fluorine to more than offset the inductive effect, and bromine, with a smaller hyperconjugation effect, shows only an inductive effect. That is, the two charge models “predict” differing amounts of the two effects. This is not surprising, and the important point is that there is little to no evidence of an inductive effect beyond two bonds, even in these extreme cases with three halogens.

Data for pentane, fluoropentane, pentylamine and pentanol are shown in Figure 8. The effects at C3–C5 and their attached hydrogen atoms are negligible and warrant no further discussion. At C1 we have the traditional inductive effect, F > O > N > H. At H1 we have H > N > *F* > O. Each electronegative element also has a +R hyperconjugation effect, and perhaps we are seeing the subtle interplay of these two effects at two bonds. We are reluctant to overanalyze any apparent trends, as another charge model will allow us to reach the same general conclusion but with slightly different relative contributions. Intuitively, we might have expected N to be most electron-donating, having a smaller inductive effect and a larger hyperconjugation effect. At C2 we have H > N > O > F, which is broadly similar.

**Figure 8:**
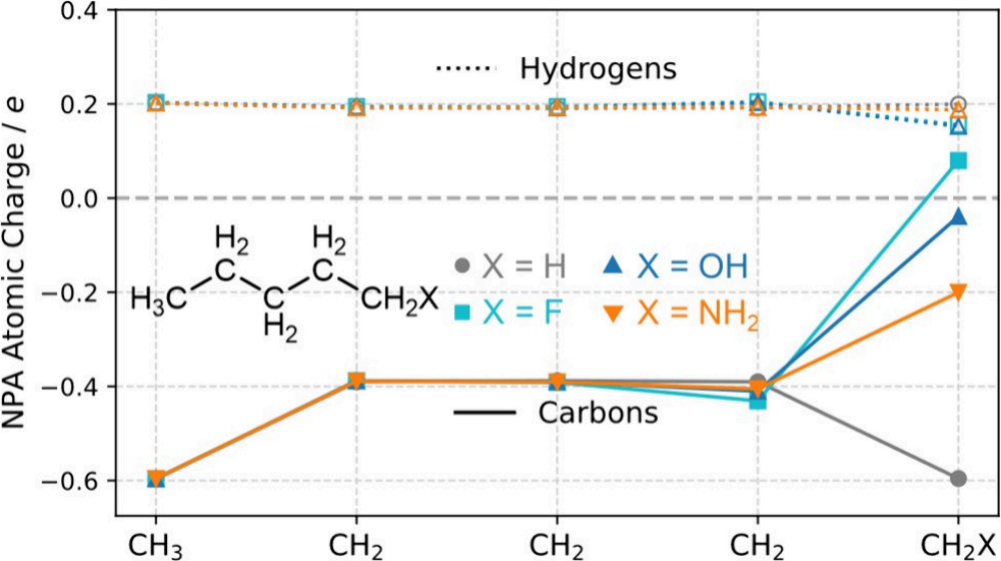
NPA atomic charges of 1-substituted pentanes. Carbon (solid lines) and hydrogen (dotted lines).

Therefore, with the most common electronegative elements found in neutral organic compounds, we find that there is no meaningful electronic effect beyond two bonds, and beyond one bond the trends are small and are complicated by hyperconjugation. This is entirely in line with the work by Wiberg,^57^ and by Nolan and Linck,^58^ and inconsistent with the inductive effect in neutral molecules as presented in textbooks. The attenuation of the inductive effect by a ‘fixed’ factor by each successive bond is not apparent from the data above. Furthermore, our conclusions are entirely in line with ^1^H NMR chemical shift data presented by Cao and Cao^44^ which show that in representative compounds CH_3_CH_2_CH_2_CH_2_X the terminal CH_3_ group is unaffected by X, and there is little difference between the 2-CH_2_ and 3-CH_2_ chemical shifts.

#### Aromatic Compounds

We will briefly compare transmission of electron-density in a benzene ring with that in an alkane. Calculated charges for the carbon atoms in four representative compounds are shown below (Figure 9).

**Figure 9:**
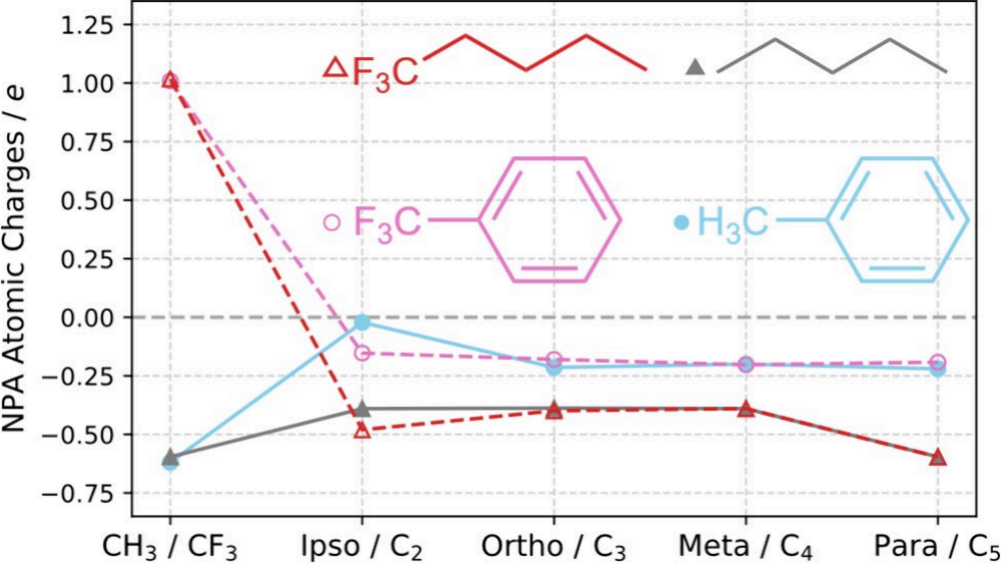
NPA atomic charges of trifluoro- and nonsubstituted pentanes and toluenes.

The charge on the CH_3_/CF_3_ carbon itself is essentially identical whether attached to sp^3^ or sp^2^ carbon.^60^ At the next carbon (*ipso*/C2) the CF_3_ group exerts a net electron-donating effect compared to CH_3_ in both the aliphatic and aromatic compounds, which is not expected based on the textbook transmission of an inductive effect. The effect is marginally larger in α,α,α-trifluorotoluene, but we would not attempt to meaningfully distinguish these two systems. While these results do not preclude a –I inductive effect at this position, they require the +R hyperconjugation effect from the fluorine lone-pairs to be larger. For the remaining positions, the differences between toluene and α,α,α-trifluorotoluene are miniscule.

### Charge Stabilization by an Inductive Mechanism

It is evident from the data of Nolan and Linck^58^ and from Wiberg^57^ that transmission of electron-density along an alkyl chain behaves differently in charged molecules than in neutral molecules.^74^ We now extend these studies with halocarboxylic acids that are ubiquitous in organic chemistry textbooks.

In Table 1 we give the calculated Δ*H* for the ionization of the various carboxylic acids. In the absence of a halogen, the longer chain carboxylic acids are found to be intrinsically more acidic. This is due to the higher polarizability of the longer alkyl groups acting to stabilize the conjugate base.

**Table 1:** Calculated Ionization Enthalpies (kJ mol^–1^) for Selected Carboxylic Acids X­(CH_2_)_n_CO_2_H

	*n* =	X = H	X = Cl	X = F
Acetic	1	1451.4	1395.8	1408.2
Propanoic	2	1449.6	1406.8	1416.6
Butanoic	3	1447.6	1419.4	1425.4
Pentanoic	4	1444.2	1426.4	1430.4
Decanoic	9	1442.8	1433.7	1438.0

For the halocarboxylic acids, the halogen enhances the acidity in line with data presented earlier (Figure 2) but has less impact further from the carboxylic acid. Contrary to expectation from the relative electronegativities of Cl and F, chloroacids are always calculated to be more acidic than the corresponding fluoroacids.

We believe this can be explained very simply. Polarizability of the halogen is more important than electronegativity. Figure 10 shows the amount of negative charge gained by X (H,F,Cl) and by (CH_2_)_n_ upon deprotonation. Upon formation of the carboxylate anion, Cl gains more electron density than F for the same length carboxylic acid. The difference between F and H is very small indeed. The amount of charge gained by the alkyl chain is larger for the longer-chain carboxylic acids, and is larger for F and H than for Cl. This means that the classic textbook picture of enhancement of acidity of halocarboxylic acids due to the inductive effect of the halogen is incomplete. The two effects (alkyl polarizability and halogen polarizability with some contribution from electronegativity) operate in a complementary manner when charge stabilization is considered. Polarizability is more important than electronegativity.

**Figure 10:**
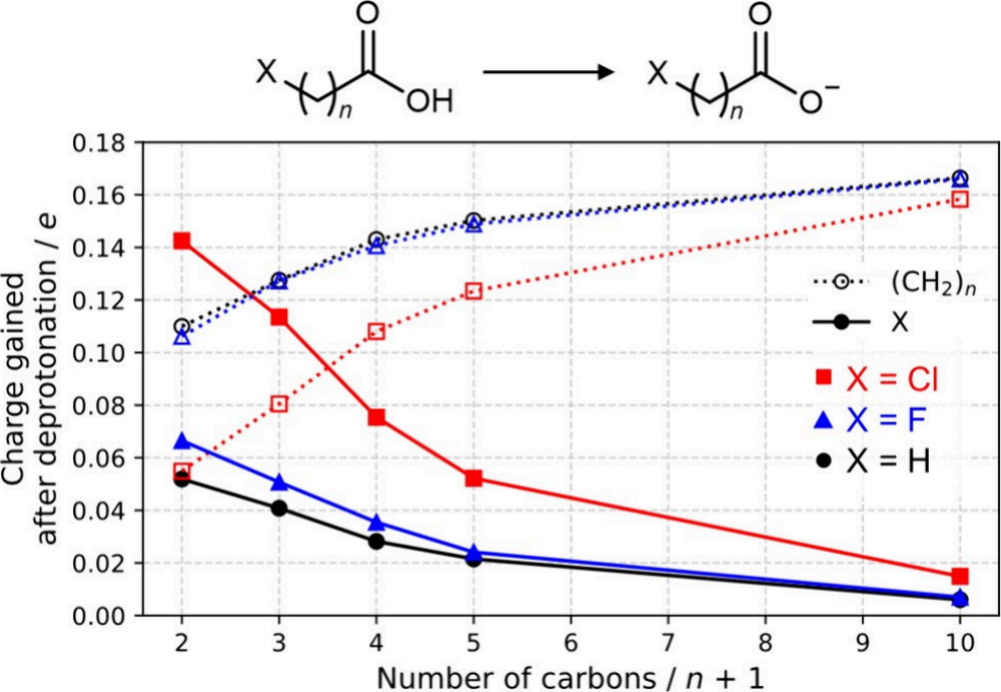
Change in NPA atomic charges of fluoro-, chloro- and nonsubstituted carboxylic acids. Dashed symbols show changes in charge of the alkyl chain (CH_2_)_*n*_ with solid lines showing the change in the charge of the substituent (X = H, F, Cl).

For aqueous p*K*_a_ values, the effects will be dominated by solvation, and we are unlikely to observe a measurable effect for 10-chlorodecanoic acid. Nevertheless, since both polarizability and the inductive effect are covered, to some extent, in all undergraduate organic chemistry textbooks, it is important that we use the right explanations, or at the very least we know when and how we are ‘simplifying the story’. Critically, the fact that the effect diminishes with distance (between the halogen and the carboxylate) does not imply that the magnitude of the effect diminishes along the chain at each additional atom. It has been established that polarization affects the extremities of a molecule much more than the interior,^75^ although the partitioning of charge between alkyl and halogen shown above makes this difficult to show clearly with these systems. We will therefore return to this point after a brief discussion of the field effect.

### Consideration of the Field Effect

Carboxylic acid **1a** is more acidic than **1b**, which in turn is more acidic than **1c** (Figure 11).^76^ This is the classic example of the field effect^77^ and was attributed to a through-space effect based on the expectation that a through-bond effect would diminish in an identical manner for **1a** and **1b** with no directional element.^16^ This is indicative of the thinking at the time.

**Figure 11:**
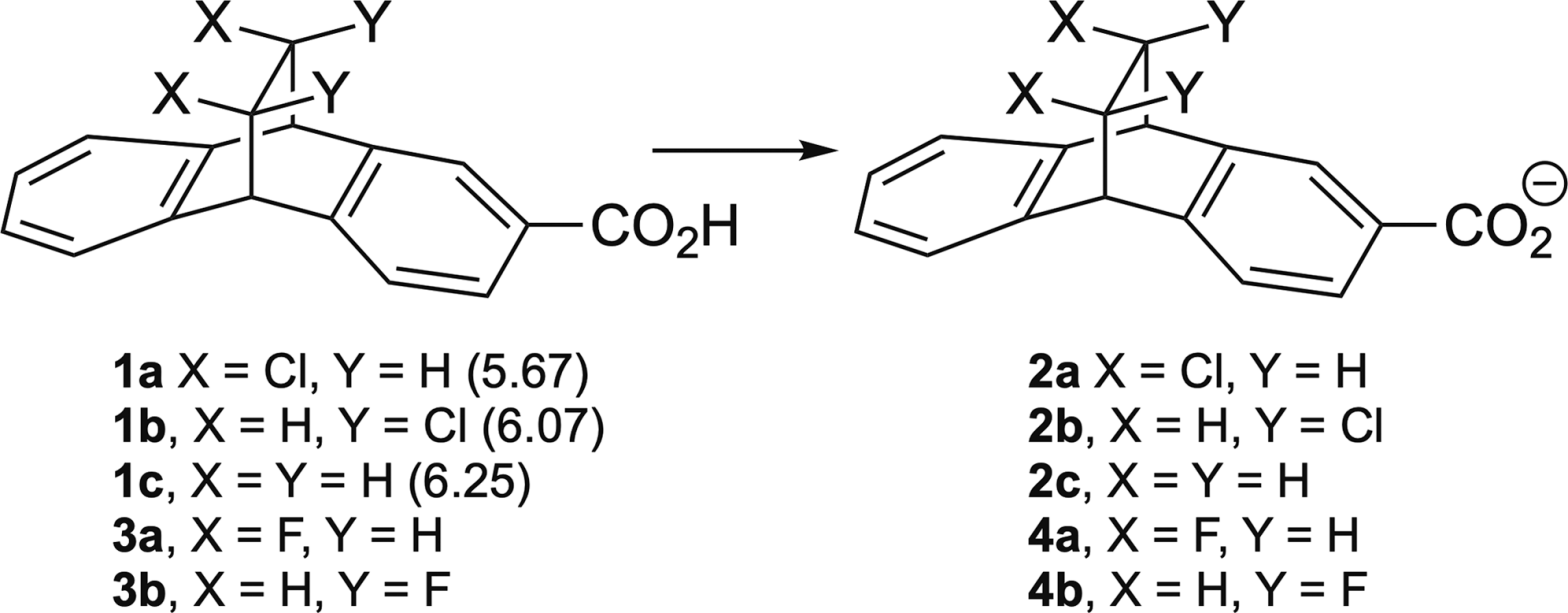
Compounds **1a** – **1c** and **3a** – **3b** with known p*K*_a_ values (50% aqueous ethanol) shown in parentheses.

In the gas phase, compound **1a** is calculated to be more acidic than compound **3a**, while **1b** is more acidic than **3b** (data in Supporting Information). This is not consistent with a through-space effect in which the carboxylate charge interacts with the C–X (X = Cl, F) bond dipole, but it is consistent with the above results in which the stabilization of the carboxylate anion can be viewed in terms of geometry-dependent polarization of the molecular orbitals in which Cl is more polarizable than F. The molecular orbitals of the carboxylate anion (Figure 12 shows one of the MOs of **2a**) span the entire molecule, so it is reasonable that the orientation of the C–Cl bonds will impact the carboxylate group. By considering the increased acidity of compounds **1a** and **1b** relative to **1c** as a polarization of the molecular orbitals, we no-longer need to invoke a through-space effect. Following our previous work,^38^ it is already necessary to revise pedagogies of ‘simple’ halocarboxylic acid acidity. This revision also provides a consistent explanation for the relative acidity of compounds **1a–c** and **3a–b**.

**Figure 12:**
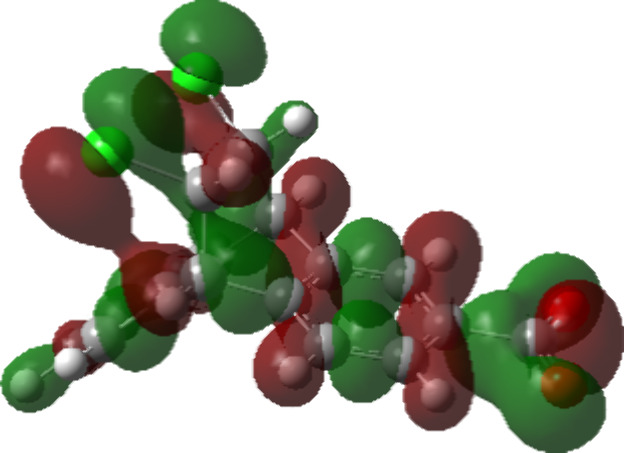
One of the molecular orbitals of anion **2a** showing that the orbital spans the molecule so that the charge on the carboxylate group will inevitably perturb the C–Cl bonds and *vice versa*.

The importance of changing our epistemology from bond-centric to an orbital-centric perspective comes from the work of Topsom.^78,79^ We have calculated the structure and charges shown in Figure 13 with *d* = 3.5 Å (the energetics of these systems according to distance is given in the Supporting Information). Upon protonation of the amine, methane gains a net positive charge of +0.003 e, while fluoromethane gains a charge of +0.007 e. We would expect fluoromethane to be less electron-donating, which we see with Hirshfeld charges (methane, + 0.021 e; fluoromethane, + 0.014 e). While different charge models can give different results, the key message is that transfer of ‘part of an electron’ between separate molecules does not fit with a classical view but is perfectly in line with a quantum mechanical molecular orbital effect. The orbitals of methylammonium and methane/fluoromethane extend (in principle) to infinity, and the existence of orbitals that span the entire system is expected.

**Figure 13:**
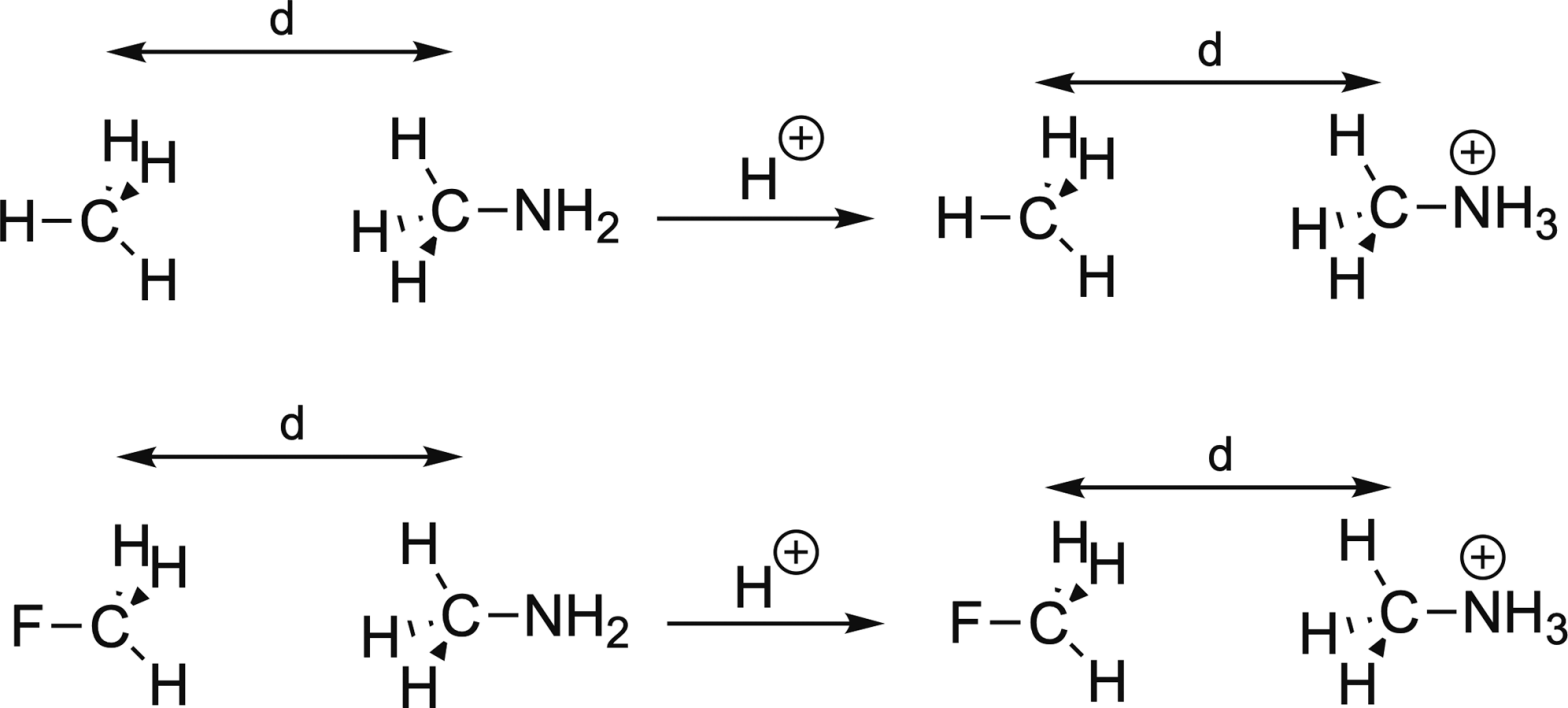
Protonation of methylamine with either methane or fluoromethane constrained at a distance ‘d’ varying from 3 to 10 Å.

We might still consider this to be a through-space effect, *but only because the orbitals of the system extend into this space*. We suggest that viewing both examples as through-orbital polarizability effects creates an opportunity to make the foundations of the subject more coherent.

### Exploring the Interface of the Inductive Effect and Polarizability

One can computationally apply polarization (via a simulated electric field) to a molecule, and to measure the effect of this polarization. This provides a bridge between the inductive and polarizability effects that would not be accessible experimentally. A calculated field of 0.005 a.u. and 0.02 a.u. was applied to 1-fluoropentane, with electron-density directed toward (+) or away (−) from F. Calculated charges are shown in Figure 14 (charges for CH_3_ and CH_2_ groups combined into a single data point). With a field of +0.02 a.u. (i.e., polarizing electron-density toward F) the impact is felt most strongly at the extremities,^75^ with only small changes in charge at the intervening atoms (red). With the field in the opposite direction (−0.02 a.u.; dark blue) electron density is notionally being transferred from F to the CH_3_ group. With fields of 0.005 a.u. in either direction (dotted lines) the effects are similar but smaller. The extent of charge transfer is not identical with the field in different directions since it is easier for the applied field to reinforce than oppose the existing polarization of the C–F bond.

**Figure 14:**
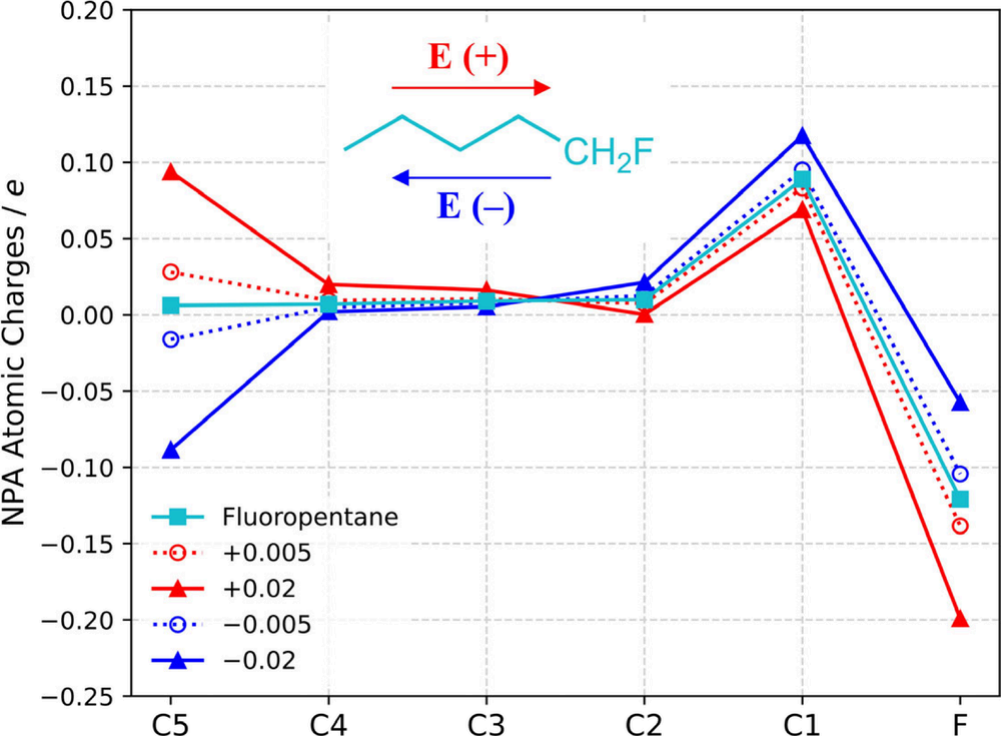
Amalgamated NPA charges (e) for 1-fluoropentane. A positive field is designated as polarizing electron-density toward F. Field strength is in a.u..

We have seen that the inductive effect in a neutral molecule is not attenuated by a factor by each successive C–C bond. We noted that it was difficult to demonstrate this clearly when polarizability was the dominant factor in charge stabilization. However, when we induce polarization computationally in a neutral molecule, we see a similar effect, in which there is net polarization of the molecule rather than progressively diminishing polarization of bonds. The inductive effect and the polarizability effect do not behave in the way described in textbooks and in the early physical organic chemistry research literature.

## Implications for Pedagogy of the Inductive Effect

The inductive effect in neutral organic molecules is not transmitted along the chain beyond the point of attachment in the manner implied by Figure 1. We suggest that this diagram should be deprecated in favor of a short-range effect.

It is well-known that a CF_3_ group is *meta* directing in aromatic electrophilic substitution reactions. The fact that a CF_3_ group is either electronically neutral or slightly inductively electron-donating in the neutral aromatic compound is not in conflict with this observation. The CF_3_ group is expected to affect the relative stabilities of the positively charged Wheland intermediates during the substitution reaction rather than any inductive effect in the aromatic substrate.

When considering stabilization of carboxylate anions by halogen substituents, a diagram such as Figure 15 appears in several textbooks,^10,19,20,80,81^ in which the δ+ on the carbon is considered to stabilize the δ− charges on oxygen, while the δ− on chlorine provides a destabilizing effect. The carbon is closer and so this interaction is considered to dominate.

**Figure 15:**
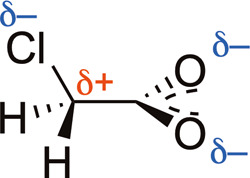
Diagram sometimes used in support of the stabilization of acetate anions by C–Cl bonds.

Essentially, this is describing the field effect,^77^ and we would expect the corresponding fluoroacetate to be more stabilized by this mechanism. This is not the case. We recommend that diagrams such as this should not be used.

The ‘underlying’ acidity trends in halocarboxylic acids are not actually reflected in solution p*K*_a_ data. However, we already deal with a similar problem in teaching the trend in acidities of simple alcohols in solution (MeOH > EtOH > *i*-PrOH > *t*-BuOH). This is the opposite of the structural (gas phase) trend. Most good textbooks cover the gas phase trend and explain that solvation is the dominant factor in delivering the solution trend and that alkyl group polarizability is responsible for the gas phase trend.^82,83^ We recommend the same approach with halocarboxylic acid acidity. The solvent effect (smaller anion is better solvated) is the same for the carboxylic acid and alcohol series, which allows for a level of consistency.

Carboxylate charge stabilization is better described as an electronegativity-enhanced polarizability effect. The importance of making the distinction between inductive and polarizability effects is seen in the fact that chlorocarboxylic acids are intrinsically more acidic than the corresponding fluorocarboxylic acids. This means that polarizability must become more prominent in organic chemistry teaching than is often the case. Since polarizability is important, the enhancement of alcohol and carboxylic acid acidity by a halogen should only be presented in the context of alkoxide and carboxylate stabilization, and not in terms of polarization of the neutral precursor bond (e.g., an electronegative atom polarizing an O–H bond to ease proton removal) as it is sometimes presented.^84–86^

The field effect is not widely taught in undergraduate chemistry courses but is included in advanced organic chemistry textbooks.^16,87^ The notion of a field effect being due to through-space interaction of a charge with a bond dipole directly contradicts our recent^38^ and new (herein) computational results on halocarboxylic acids. The classic examples of the field effect (Figure 11) are entirely consistent with the simpler halocarboxylic acids. Both can be considered as polarizability effects. The field effect may not be needed, as has been suggested elsewhere.^88^ This creates an opportunity for the inclusion of these interesting molecules in curricula.

In more advanced study, connections with orbital effects can be made. Polarizability effects are whole molecule orbital effects in which the molecular orbitals are being polarized by a substituent. Polarization affects the extremities of the molecular orbitals more than the middle,^75^ so there is no reason to believe that polarizability is attenuated with each passing bond in the way that is commonly taught.

## Conclusions

Many of the problems associated with the pedagogy and common understanding of the inductive effect relate to an expectation of how an inductive effect *should* behave. The notion of electron transmission through localized bonds has permeated this area. Many of the pioneers of the discipline, working in very different times, had expectations for how electron-transmission would take place, and when those expectations did not match with reality, additional effects (such as the field effect) were introduced.

The present study applies modern computational methods to the inductive effect. We see that the inductive effect in neutral molecules is short-range, while polarizability effects are more important when it comes to explaining diverse effects such as carboxylic acid and alcohol acidity, or leaving group trends in substitution reactions. The situation is compounded by the fact that according to the IUPAC definition, the polarizability effect *is* an inductive effect, and we respectfully suggest that an opportunity exists to clarify this situation.

This work, which has implications at almost all levels of organic chemistry study, gives educators a more complete picture of the inductive and associated electronic effects. It is important that our teaching reflects the latest research data. After all, stating that trifluoroacetic acid is more acidic than trichloroacetic acid because F is more electron-withdrawing than Cl is simply incorrect.

## Supplementary Material



## Data Availability

The computational data supporting the results presented in this article are freely available via the Cardiff University Data Catalogue (10.17035/cardiff.31168435).

## Notes

Graphs showing alternative charge decomposition models (Hirshfeld, CM5) (PDF)
