# Supplementary material for: Rethinking the Nature and Extent of Inductive Effects in Organic Compounds
Source: J Chem Educ. 2026 May 14;103(6):3156–64. doi: 10.1021/acs.jchemed.6c00141 (PMC13262726; doi:10.1021/acs.jchemed.6c00141)
Supplement: Supplementary file 1 [file ed6c00141_si_001.pdf]

Rethinking the Nature and Extent of Inductive Effects in Organic Compounds

Mark C. Elliott,[a] Edwin C. Johnson,[b] Kasimir P. Gregory[c] and Colan E. Hughes[a]

[a] *School of Chemistry, Cardiff University, Park Place, Cardiff, CF10 3AT, UK.*
[b] *College of Science, Engineering and Environment, University of Newcastle, Callaghan, 2308, NSW, Australia*
[c] *School of Science and Technology, University of New England, Armidale, NSW 2351, Australia*

General Points

In all cases, geometries were optimized, and frequency calculations were done to confirm minima and obtain thermochemical corrections. Comprehensive conformational searches were not routinely conducted, with 'extended zig-zag' conformers of alkyl chains being used as the starting point for geometry optimization. This has the advantage that the structures are directly comparable.

Consideration of Other Charge Models

The supporting information consists mostly of consideration of other charge models. These are organised in the same way as the original manuscript.

Figure S1 corresponds to **Figure 5** in the manuscript.

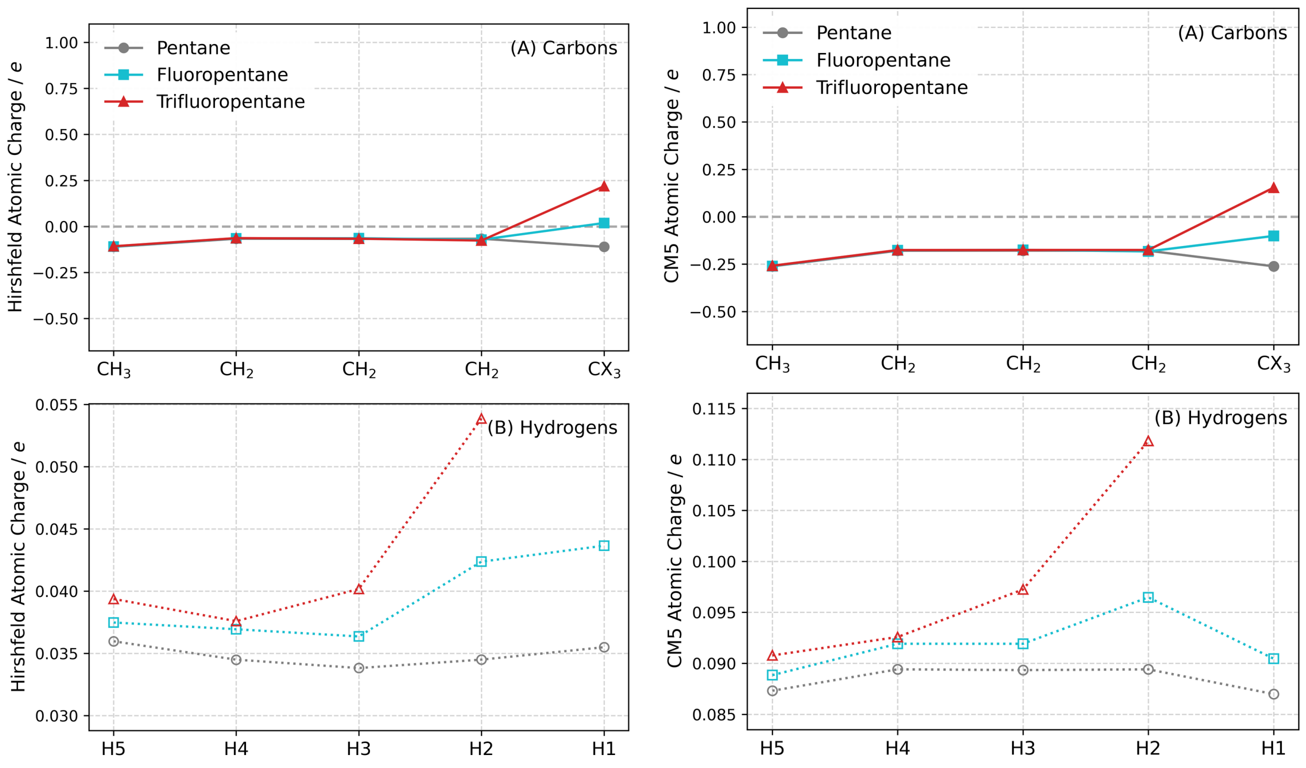

Figure S1. (a) Hirshfeld (left) and CM5 (right) Charges on Hydrogen and Carbon in Pentane, 1-Fluoropentane and in 1,1,1-Trifluoropentane; (b) Expansion of the same charges on the hydrogen atoms along the pentane chains.

As we saw with the NPA charges, we have no effect beyond C2, and a small electron-donating effect at C2 itself. While NPA charges predicted electron-donation to H1, the Hirshfeld and CM5 charges do not. However, in all cases the effect at H is very small.

Figure S2 corresponds to **Figure 7** in the manuscript.

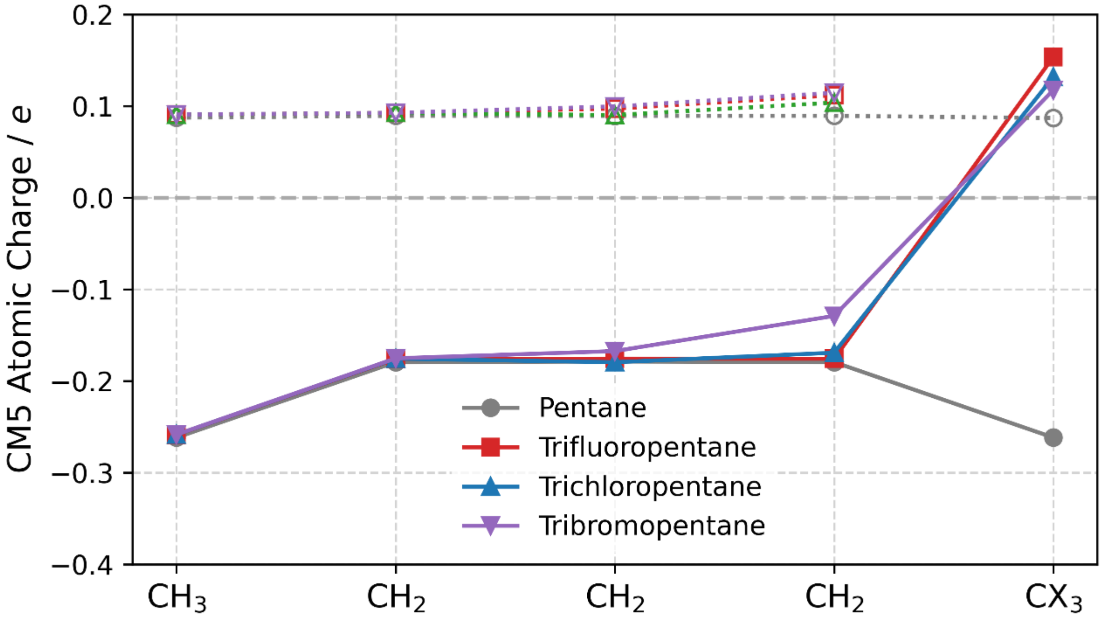

Figure S2. CM5 atomic charges of trihalopentanes. Carbon (solid lines) and Hydrogen (dotted lines).

These closely parallel the Hirshfeld charges discussed in the paper.

Figure S3 corresponds to **Figure 8** in the manuscript.

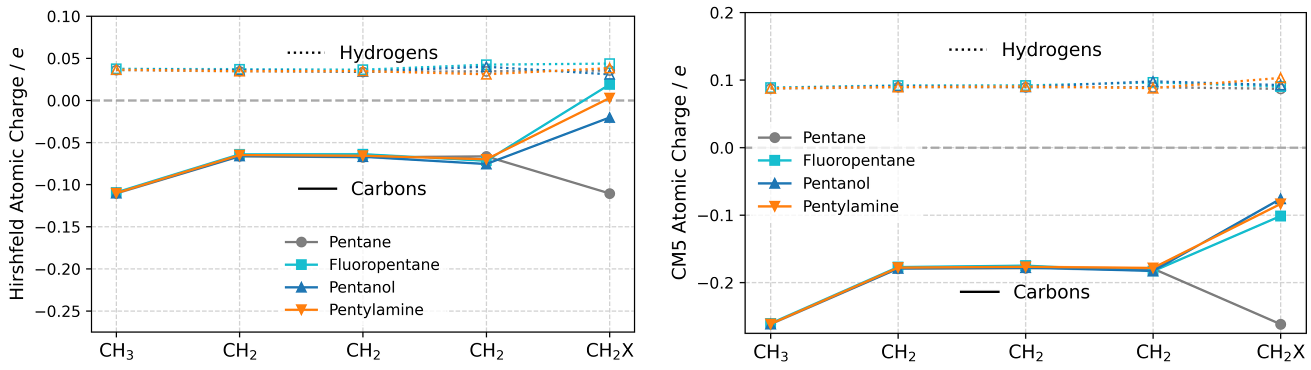

Figure S3. Hirshfeld (left) and CM5 (right) atomic charges of 1-substituted pentanes. Carbon (solid lines) and Hydrogen (dotted lines).

Effects at C2 are small and it is difficult to define a trend. There is no effect beyond C2.

Figure S4 corresponds to **Figure 9** in the manuscript.

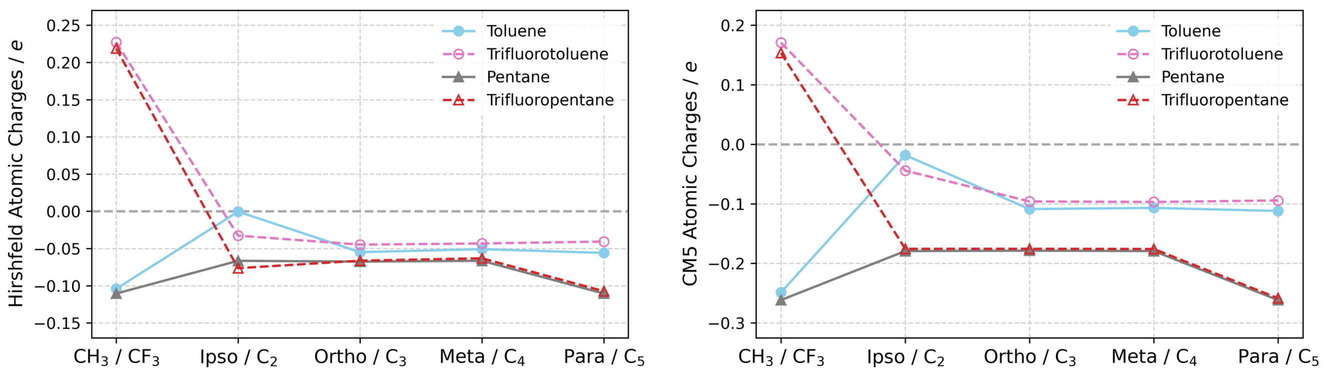

Figure S4. Hirsfeld (left) and CM5 (right) atomic charges of trifluoro- and non-substituted pentanes and toluenes.

The trends here mirror those with NPA charges (main manuscript), but the hyperconjugation effect at C2 in trifluoropentane is smaller than in trifluorotoluene.

Figure S5 corresponds to **Figure 10** in the manuscript.

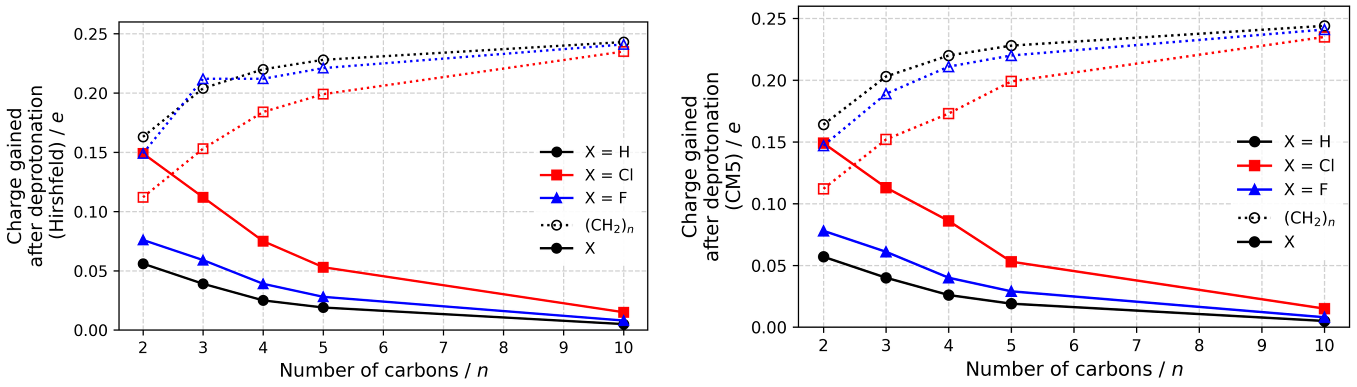

Figure S5. Change in Hirshfeld (left) and CM5 (right) atomic charges of fluoro-, chloro- and non-substituted carboxylic acids. Dashed symbols show changes in charge of the alkyl chain $(CH_2)_n$ with solid lines showing the change in the charge of the substituent (X = H, F, Cl).

With the Hirshfeld charges, 3-fluoropropanoic acid is a bit of an outlier here, but the CM5 charges, which are related to Hirshfeld, look fine. These data have been checked and this is reproducible. Nevertheless, all charge models are in close alignment.

The Field Effect

We have calculated the dissociation enthalpies of compounds **1a – 1c**, giving carboxylates **2a – 2c**, as well as the fluorinated analogues **3a** and **3b** giving carboxylates **4a** and **4b**.

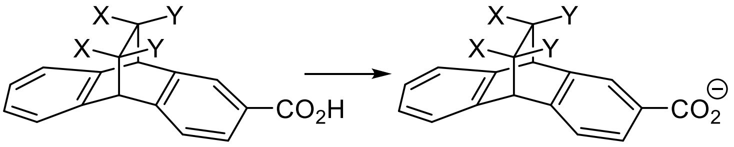

1a X = Cl, Y = H
1b, X = H, Y = Cl
1c, X = Y = H
3a, X = F, Y = H
3b, X = H, Y = F

2a X = Cl, Y = H
2b, X = H, Y = Cl
2c, X = Y = H
4a, X = F, Y = H
4b, X = H, Y = F

Figure S6. Bicyclic carboxylic acids and carboxylates used to exemplify the field effect

The thermodynamic data are given below (**Table S1**).

Table S1: Dissociation enthalpies for carboxylic acids **1** and **3**.

Carboxylic Acid	Dissociation Enthalpy/kJ mol^{-1}
**1a**	1385.0
**1b**	1403.3
**1c**	1412.9
**3a**	1387.5
**3b**	1406.4

The key observation is that acidity Cl > F > H in both cases, which would not be expected based on the 'traditional' field effect. However, this is perfectly logical based on polarizability.

Charges on the halogens, and amalgamated charges for the carboxylic acid/carboxylate groups are shown in the structures (**Figure S7**).

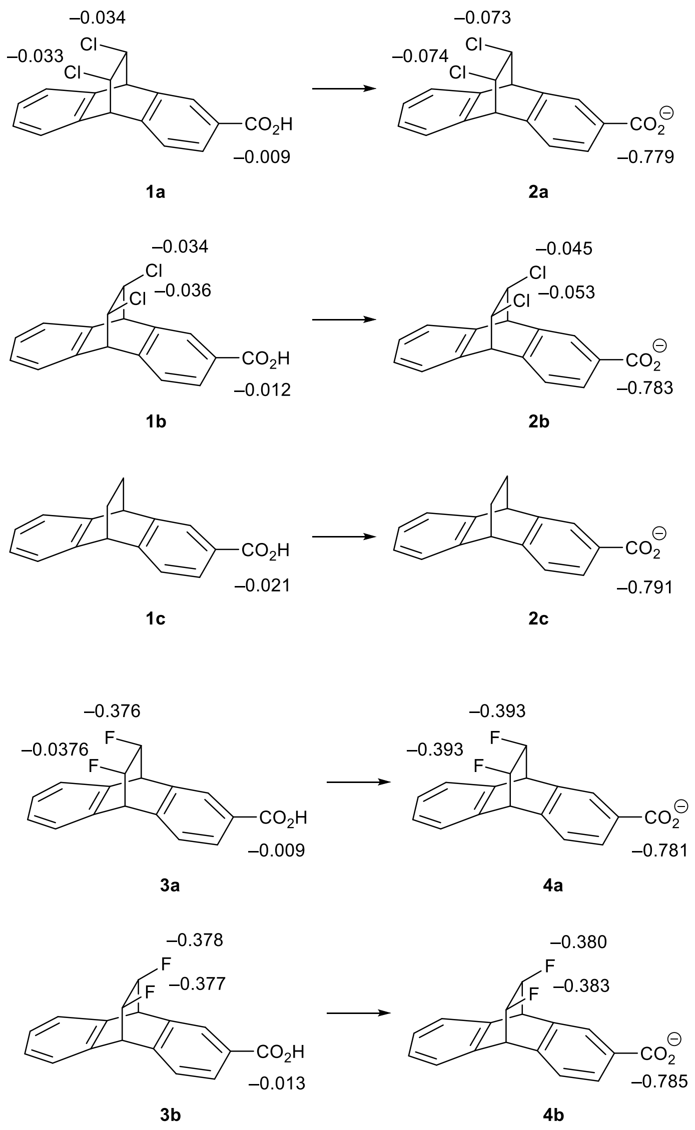

Figure S7. Calculated NPA charges for structures **1 – 4**.

Upon deprotonation, Cl gains more negative charge than does F, although naturally F has a larger partial negative charge than Cl in both the carboxylic acid and the carboxylate.

Through-Space Field Effects Between Molecules

In the manuscript we discussed the following example (**Figure S8**), in which we showed that methane or fluoromethane donate fractional electron-density to a proximal methylammonium molecule.

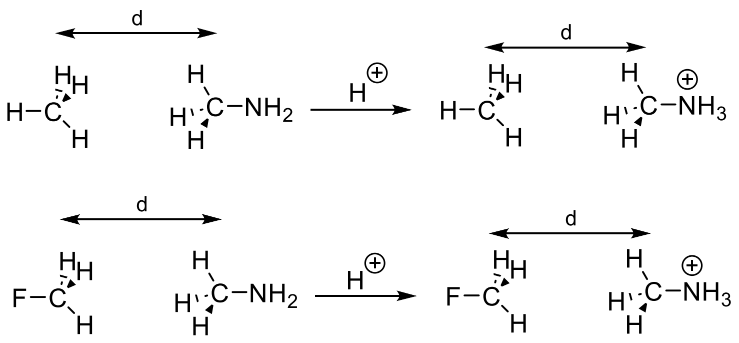

Figure S8. Protonation of Methylamine with Either Methane or Fluoromethane Constrained at a distance 'd' Varying from 3 Å to 10 Å.

Here, we provide additional information. In **Figure S9** we show the relationship between the distance (d, Å in 0.1 Å steps) and relative energy for all four species in **Figure S8**. The proximity (between 3 Å and 10 Å) of methane to methylammonium results in a net stabilization throughout this range. Conversely, the proximity of fluoromethane destabilizes methylammonium across this range. The proximity of either methane or fluoromethane to methylamine behaves similarly, providing a stabilization in the first instance (to d = 3.6 Å) which then becomes destabilizing. As a result, the proximity of methane renders methylamine more basic across the range investigated, while the proximity of fluoromethane renders methylamine less basic. This is in good agreement with Topsom's data as cited in the main manuscript.

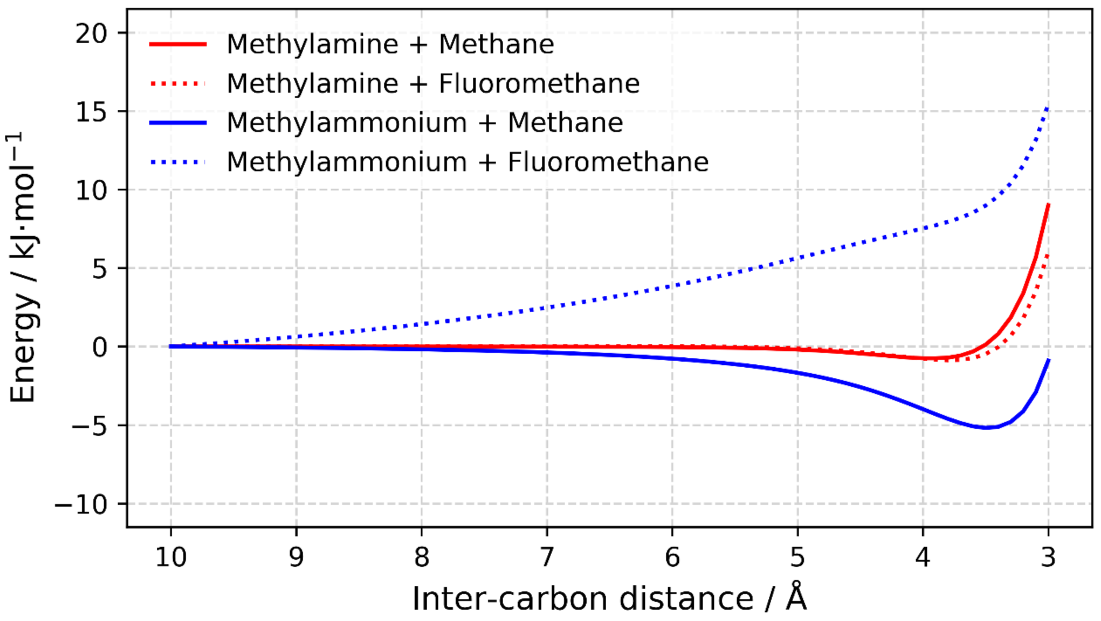

Figure S9. Relative Energies (kJ mol^{-1}) of Each System as a Function of Inter-Carbon Distance (d).

Figure S10 corresponds to **Figure 14** in the manuscript**.**

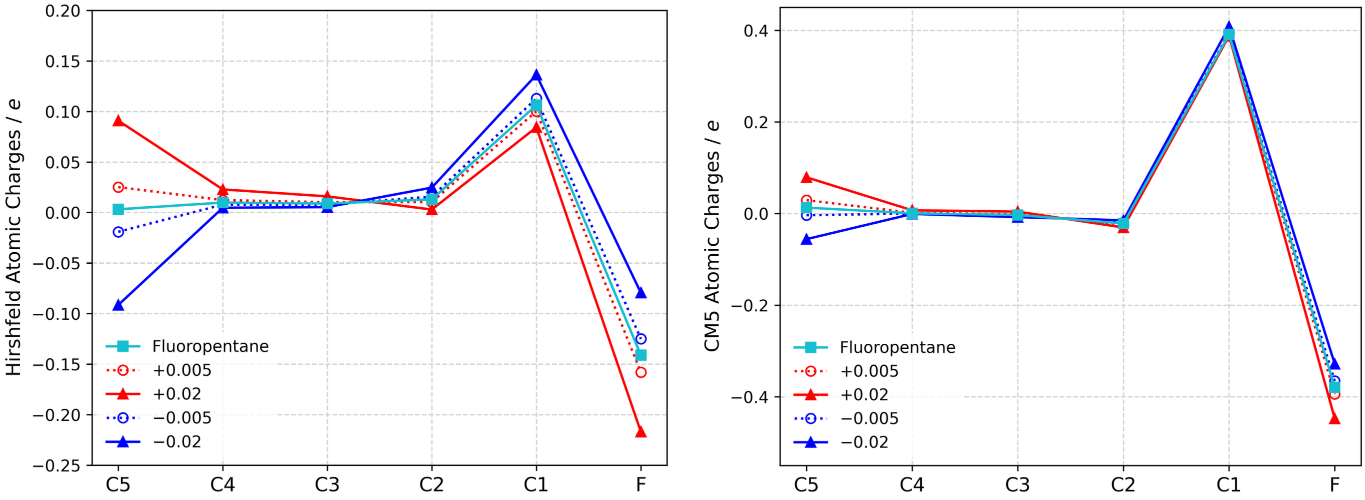

Figure S10. Amalgamated Hirshfeld and CM5 charges (e) for 1-fluoropentane. A positive field is designated as polarizing electron-density towards F.

While there are small differences to the individual charges, all charge models are in extremely good alignment.
